# Individual-Level and Couple-Level Chronic Condition Discordance: Longitudinal Links to Perceived Control

**DOI:** 10.1093/geroni/igaa057.2234

**Published:** 2020-12-16

**Authors:** Courtney Polenick, Kira Birditt, Angela Turkelson, Sadie Shattuck, Helen Kales

**Affiliations:** 1 University of Michigan, Ann Arbor, Michigan, United States; 2 UC Davis, Sacramento, California, United States

## Abstract

Chronic condition discordance (i.e., the extent that two or more conditions have non-overlapping self-management requirements) has adverse mental health implications but little is known about mechanisms accounting for these links. We considered how chronic condition discordance at the individual level and the couple level (i.e., between spouses) was associated with perceived control among 2,676 couples from three waves (2006, 2010, and 2014) of the Health and Retirement Study. Dyadic growth curve models revealed that when wives had greater individual-level discordance, they reported lower initial health-related control and personal mastery and steeper reductions in personal mastery. When husbands had greater individual-level discordance, they reported lower initial health-related control and faster declines in health-related control and personal mastery, and wives had faster declines in personal mastery. When there was greater couple-level discordance, wives reported lower initial health-related control. Targeting increases in perceived control among older couples managing complex conditions may be beneficial. Part of a symposium sponsored by Dyadic Research on Health and Illness Across the Adult Lifespan Interest Group.

